# Implementing a Compression Technique on the Progressive Contextual Excitation Network for Smart Farming Applications

**DOI:** 10.3390/s22249717

**Published:** 2022-12-12

**Authors:** Setya Widyawan Prakosa, Jenq-Shiou Leu, He-Yen Hsieh, Cries Avian, Chia-Hung Bai, Stanislav Vítek

**Affiliations:** 1 Department of Electronic and Computer Engineering (ECE), National Taiwan University of Science and Technology, Taipei 106335, Taiwan; 2 Faculty of Electrical Engineering, Czech Technical University in Prague, Technicka 2, 16627 Prague, Czech Republic

**Keywords:** deep learning, model compression, progressive contextual excitation, pruning filters

## Abstract

The utilization of computer vision in smart farming is becoming a trend in constructing an agricultural automation scheme. Deep learning (DL) is famous for the accurate approach to addressing the tasks in computer vision, such as object detection and image classification. The superiority of the deep learning model on the smart farming application, called Progressive Contextual Excitation Network (PCENet), has also been studied in our recent study to classify cocoa bean images. However, the assessment of the computational time on the PCENet model shows that the original model is only 0.101s or 9.9 FPS on the Jetson Nano as the edge platform. Therefore, this research demonstrates the compression technique to accelerate the PCENet model using pruning filters. From our experiment, we can accelerate the current model and achieve 16.7 FPS assessed in the Jetson Nano. Moreover, the accuracy of the compressed model can be maintained at 86.1%, while the original model is 86.8%. In addition, our approach is more accurate than ResNet18 as the state-of-the-art only reaches 82.7%. The assessment using the corn leaf disease dataset indicates that the compressed model can achieve an accuracy of 97.5%, while the accuracy of the original PCENet is 97.7%.

## 1. Introduction

Deep learning (DL) has been widely acknowledged as a superb scheme for computer vision tasks as a pattern recognition technique. The superiority of deep learning has been inevitably recognized in recent years. This has also encouraged the study of the deep learning approaches used in several fields. For instance, most computer vision tasks use DL approaches to accomplish the goal, particularly for object detection and image classification [1,2,3]. Furthermore, DL is also used for a range of scenarios, such as malicious detection schemes [4] and even the creation of synthesis images [5].

As one of the potential fields for DL applications, smart farming applications based on deep learning techniques are widely proposed by researchers and further applied by the industry. Fine-grained classification tasks, pesticide prediction, and plant disease prevention applications are the areas that researchers study to implement the DL approaches. In our recent study [1], we also assessed whether the utilization of the DL technique could even beat the performance of conventional methods. In our first attempt [6], the gray-level co-occurrence matrix (GLCM) method was used to perform the classification task on cocoa bean images. Then, our further study shows that by implementing the feature enhancement using the attention mechanism, we can enhance the classification performance presented by [6] to achieve more accurate classification models.

Even though DL is regarded as the superior technique for several applications, the complexity of the computation and matrix calculation performed by DL techniques becomes the main issue for deployment. Most DL models are often considered too big and complicated to calculate. Therefore, the running time is very slow, especially without additional special hardware. Thus, to tackle this issue, engineers can employ high-end hardware such as GPUs or TPUs. However, it is not acceptable if the DL technique is used for one or two specific tasks, since the cost of investment using this special hardware may be heavily burdened. Therefore, several attempts to reduce the size of DL techniques are proposed and studied. Mostly, the compression techniques applied for deep learning models are the use of pruning schemes [7,8], restructuring the existing network [9,10], quantizing [11] the weight of the deep learning models, and employing knowledge distillation schemes [12].

Restructuring the existing models aims to provide a lighter model than the existing one. The effort to create a light model is shown by the proposal by [9] that presents MobileNet as the network structure for mobile applications. SqueezeNet [10] is also one example of attempts to construct a light deep learning model. However, using this approach is suitable if we have sufficient infrastructures to conduct training, since this effort may need several trials to obtain a final model that fits the requirements of the tasks. Mostly using this technique, we need a better understanding to design a new deep learning model. In addition, it may take time to achieve an acceptable result.

If our infrastructure is insufficient, we can leverage a simple yet effective method to create and construct lighter models based on the existing model. For example, [13] presents the study of the pruning schemes for MobileNet using pruning filters. The pruning filter technique can be considered a simple methodology, but it is proven that we can compress and create a lighter network using this technique.

In this research, we will show our efforts in building a deep learning model for smart farming applications, compressing our existing model called the Progressive Contextual Excitation Network (PCENet) [1], and deploying it into the edge platform. Considering that our infrastructure is not powerful enough for implementing a complex compression methodology, and we have built the PCENet [1] as the deep learning approach for our smart farming scenarios, in this paper, we present several investigations to construct a model for the edge platform using the underlying conditions explained before, as follows.

1. We show a compression technique applied to our past effort in smart farming called the Progressive Contextual Excitation Network (PCENet).
2. We present the effectiveness of our approach to compress the existing model, accelerate the running time, and maintain accuracy.
3. We investigate the deployment of the compressed model into the edge platform and compare it with the original model.
4. We evaluate our proposed approach on real-world cocoa bean data and a publicly available dataset. Therefore, we can understand how the model works in the real captured image data.

The rest of the paper is presented as follows. The study of related work or similar attempts compared to our proposed scheme is discussed in Section 2. The illustration and description of the proposed study are elaborated in Section 3. Section 4 explains the experimental results and discusses the proposed approach compared to other existing works. The summary of the study is written in Section 5.

## 2. Related Works

Smart agriculture has been widely adopted to address several issues, such as the shortage of workers, increasing productivity, and agricultural surveillance purposes. Many use known methodologies such as computer vision, the Internet of Things (IoT), and artificial intelligence. Computer vision techniques are often used for surveillance purposes, while the IoT paradigm is often adopted along with computer vision. Furthermore, artificial intelligence (AI) schemes such as deep learning and conventional methodologies such as statistical analysis are employed to create autonomous systems.

In recent trends, deep learning-based schemes are often considered the most accurate technique to construct an AI scheme. However, some bottlenecks may occur in deploying deep learning-based schemes for agricultural sectors. For instance, a system built using deep learning techniques may need a big investment, since the computational cost of deep learning is huge. On the other hand, farmers may not need such powerful resources, and can then save the capital. Therefore, to address this issue, we need to create a deep learning scheme that is light enough to be run on a cheaper platform and suitable for agricultural sectors.

Researchers have conducted some studies to develop machine learning or deep learning models for smart agriculture. For example, ref. [14] developed a machine learning scheme using a combination of the PART classification algorithm and a wrapper feature selection scheme for an IoT-based platform applied to predict drought and crop productivity. The authors in [15] proposed the utilization of autoencoders to create a secure deep learning platform for smart farming, and ref. [16] assessed a deep learning scheme based on U-Net architecture for unmanned aerial vehicles (UAV) on Jetson Nano. In addition, ref. [17] also presented the construction of a deep learning scheme for smart agriculture to identify the biotic stress of rice crops using an edge computing paradigm. However, previous work proposed a new deep learning model and trained the network from scratch. In addition, based on our experience, it may take longer to construct and train a scheme from scratch, specifically to find and tune suitable parameters to achieve the best performance.

To enrich the study regarding constructing a smart farming model based on the optimization and compression techniques of the existing model; therefore, in this study, we proposed a compression technique applied to our smart farming model called PCENet [1] to optimize the performance and further accelerate the speed of the PCENet model. The proposed model was assessed on the cocoa bean images as the real-world problem in the Indonesian agricultural industry. To measure the generalization of the model, we also performed the performance evaluation using publicly available datasets called corn leaf disease datasets [18] and compared the accuracy of the model with the previous work [19,20].

## 3. Proposed Methodology

In this study, we discovered that the model size of our previous approach [1] is unsuitable for immediately deploying to the edge platform. Since the edge platform is not as powerful as the server-based or personal computer (PC)-based platform, we have to further compress and reconstruct our model to be lighter than the original. Moreover, we incorporated the compression technique, as presented by [7], to be implemented to compress the PCENet model. The pruning filter technique was adopted to give an insight to the practitioners that despite using a non-complicated compression scheme, we can still achieve the objective of accelerating the original model. Therefore, the compact model can be established and is suitable for deployment in edge computing.

### 3.1. Progressive Contextual Excitation

#### 3.1.1. Overview

**Figure 1:**
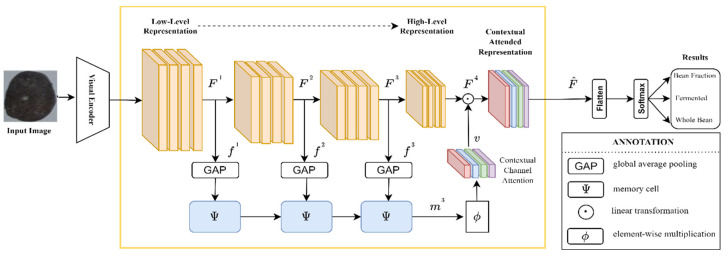
Progressive Contextual Excitation Network (PCENet).

In progressive contextual excitation (PCE), we propose to guide higher-level representation by utilizing multi-level cues. In essence, the PCE mechanism is similar to the squeeze-and-excitation mechanism. The PCE model described in Figure 1 is outlined. As a first step to defining the PCE workflow, we define the basic linear transformation of *φ* by

$$\varphi \left(x\right)=xW+b$$

where x, W, and b are each a feature input, a projection matrix, and a bias, respectively.

#### 3.1.2. Contextual Memory Cell

The exact dimensions of each representation $F^{l}\;\in \;{\mathbb{R}}^{\;h^{l}x\;w^{l}x\;c^{l}}$, where $h^{l}$, $w^{l}$, and $c^{l}$ indicate the height, width, and number of channels at each layer, respectively. The channel-wise statistics $f^{l}\;\in \;{\mathbb{R}}^{\;c^{l}}$ are calculated using a global average pooling method. To integrate the contextual channel-wise statistics, we adopt a memory cell that adaptively selects input information. Memory cells Ψ can be defined as follows:

$$i^{l}=\sigma \left(\phi \left(f^{l}\;\right)+\varphi \left(m^{l-1}\right)\right)$$

$$o^{l}=\sigma \left(\varphi \left(f^{l}\;\right)+\varphi \left(m^{l-1}\right)\right)$$

$$s^{l}=tanh\left(\varphi \left(f^{l}\;\right)+\varphi \left(i_{l}\;\cdot \;m^{l-1}\right)\right)$$

$$m^{l}=o^{l}\;\cdot \;m^{l-1}+\left(1-o^{l}\right)\;\cdot \;s^{l}$$

Input gate, output gate, candidate state, sigmoid activation function, hyperbolic tangent function, and memorized cue are designated by $f^{l},\;i^{l},\;o^{l},\;s^{l},\sigma ,\;tanh$, and $m^{l}$, respectively. In this case, the memorized cue $m^{l}$ is composed of contextual channel-wise representations to include finer-grained visual information.

#### 3.1.3. Contextual Channel Attention

As shown in Figure 1, the final contextual channel-wise representation $m^{3}\;\in \;{\mathbb{R}}^{\;c^{4/r}}$ is $m^{3}$ divided by r, where r is 16 and $c^{4}$ is 2048 empirically. We explore contextual channel-wise dependence using a linear transformation to obtain a channel-wise relationship. As a result, the contextual channel attention *v* is determined by *v* = $\left(\phi \;\left(m^{3}\right)\right)$, where *v* $\in \;{\mathbb{R}}^{\;c^{4}}$. As a result, we define the contextual attended representation as follows:

$$\hat{F_{j}}=F_{J}^{4}\;\cdot \;v_{j}$$

Assume that $F_{J}^{4}$ and $v_{j}$ correspond to high-level representation and contextual channel attention on the jth channel, respectively. For determining the category, the contextual attended representation $\hat{F}$ involves low-level visual representations with fine-grained information and is input to the following linear transformation.

### 3.2. Pruning Filters

A deep learning model needs to be designed efficiently, since each dataset probably needs a specific design to optimize the performance, avoid overfitting or underfitting problems during the training stage, and achieve an appropriate latency to complete the analysis. Therefore, a compression technique is conducted to optimize the performance of the deep learning scheme to meet a balance between accuracy and computational times.

**Figure 2:**
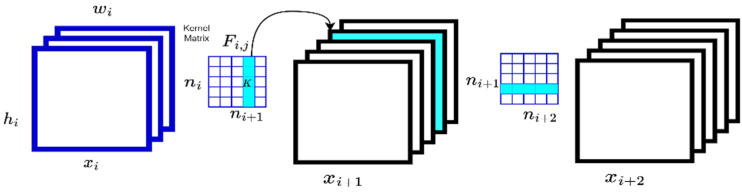
Compression technique using pruning filters.

Based on [7], pruning-based compression is a technique used to filter trained neural networks. It is possible to choose a filter that does not significantly affect the performance of the whole structure. As an example of how pruning filters can be used to reduce networks, we can refer to Figure 2.

The whole scheme begins with the input of the convolutional neural network models. In Figure 2, the input is represented by $n_{i}$. Therefore, using $n_{i+1}$ 3D filters $F_{i,j}\;\in \;{\mathbb{R}}^{\;n_{i}\;x\;k\;x\;k}$ on the $n_{i}$ input channels, then the feature map on the output side can be derived from the input features $x_{i}\;\in \;{\mathbb{R}}^{\;n_{i}\;x\;h_{i}\;x\;w_{i}\;}.$ In this case, each filter will create one feature map and is constructed by $n_{i}$ 2D kernels $K\;\in \;{\mathbb{R}}^{k\;x\;k}$. Once the pruning filter is performed on the filter $F_{i,j}$, then, as depicted in Figure 2, the corresponding feature map created by $F_{i,j}$ in $x_{i+1}$ is eliminated. Moreover, the kernels on the removed feature maps are completely deleted.

The first step in determining which filters to prune within a single layer is to calculate the L1-norm to determine the less useful filters that need to be pruned. The computation of L1-norm for each filter $F_{i,j}$ is performed by calculating the sum of its absolute kernel weights $s_{j}=\sum_{l=1}^{n_{i}}\sum \left|K_{l}\right|$. Following the calculation of the L1-norm, the filters are sorted by $s_{j}$, after which the L1-norm is calculated. As a result of pruning m filters, the filters with the smallest sum values and their associated feature maps are removed. The kernel in the next convolutional layer that corresponds to the pruned feature maps is also removed by removing the filter and corresponding feature maps. As a final step, a new kernel matrix is constructed for the *i*th and *i* + 1th layers. A new model is then developed, which incorporates the remaining kernel weights.

### 3.3. Proposed Scheme

**Figure 3:**
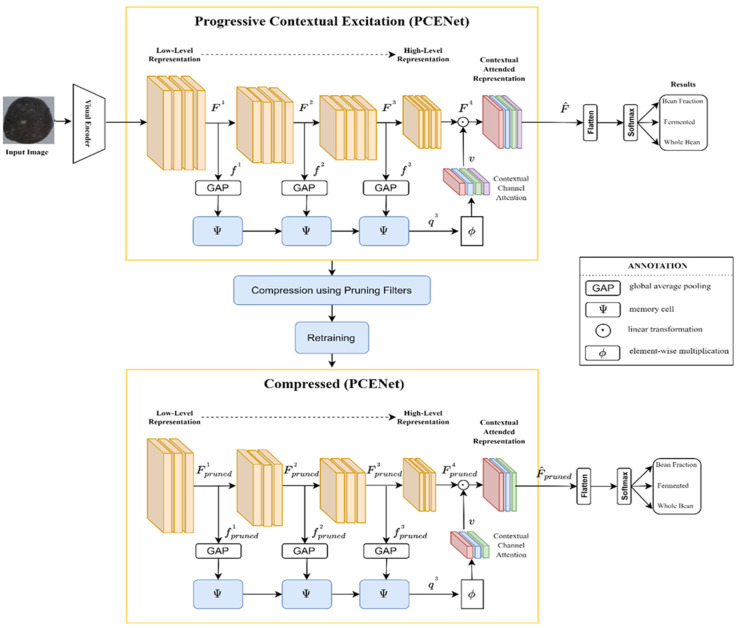
PCENet compression scheme.

In order to compress the existing PCENet model, as depicted in Figure 1, we employed the pruning filters as illustrated in Figure 2. Therefore, the combination scheme of our approach (PCENet and pruning filters) or the whole picture of our proposed scheme is illustrated in Figure 3.

As depicted in Figure 3, the PCENet model, as presented in [1], is further compressed by pruning filters. Furthermore, we retrain the model after the compressing technique is performed. Eventually, a light model is constructed and could be further assessed and deployed into the edge platform. In this study, we assessed the cocoa bean images.

Overall, the proposed scheme adds a compression scheme to reduce the complexity of the PCENet model. The compression technique is applied to prune the number of available filters on each feature. As can be seen in Figure 3, the PCENet model is constructed by the low-level feature until the high-level feature and there is the attention mechanism to leverage each level feature. Therefore, the pruning strategy is executed on each feature layer. Eventually, the lighter model can be produced by implementing the pruning filters as the compression strategy. In addition, a slimmer model, as depicted in Figure 3, is assessed and measured on the edge computing platform. The dimension of each layer on the PCENet before and after the pruning procedure is presented in Table 1.

## 4. Results and Discussion

This section discusses the experimental details and results obtained from our proposed approach and the existing deep learning techniques. This section also elaborates on the dataset used to evaluate the proposed scheme and conduct the experiment.

### 4.1. Dataset

The dataset used in this study is the cocoa bean image data obtained from South Sulawesi, Indonesia. In addition, to measure the generalization of the proposed work, we also measured the performance of the proposed work using publicly available datasets called corn leaf disease datasets [18].

**Figure 4:**
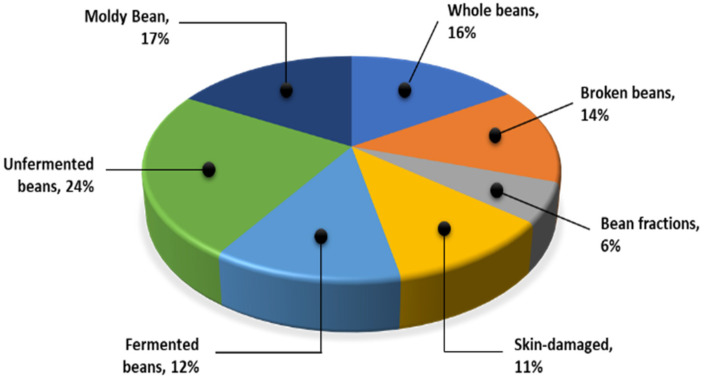
Distribution class of cocoa bean dataset.

As presented in [6], the cocoa bean data were collected using a compact digital camera and taken from the factory. In this dataset, there are 7428 images. There are seven classes: whole beans, broken beans, bean fractions, skin-damaged beans, fermented beans, unfermented beans, and moldy beans. The distribution class of the dataset is depicted in Figure 4.

**Figure 5:**
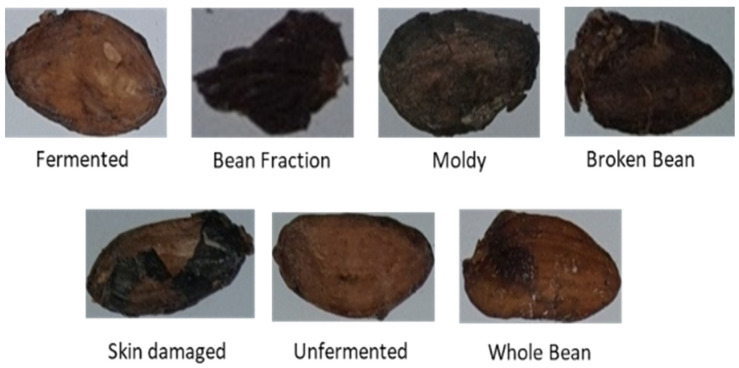
Sample images from the cocoa bean dataset.

Based on the Indonesian Standardization Institution’s regulations for exporting quality, cocoa beans are divided into seven classes. First, whole beans have a complete seed coat covering the whole seed without fractures. Another type of cocoa bean is broken beans, which have half (1/2) or less of a bean left. The next type is the bean fraction, which is less than half (1/2) of the whole bean. The fourth type of cocoa bean is a skin-damaged bean, which has a missing shell and is half the size of a whole bean. The fifth type of bean is fermented beans, which are washed or unwashed after curing and drying. Sixth, unfermented beans (e.g., slate or solid grayish blue) are composed of sliced grayish chips with a dirty white surface. Lastly, there are moldy beans, which are cocoa beans with mold on the inside, and when split open, it is easy to see the mold inside. All of these samples can be seen in Figure 5.

**Figure 6:**
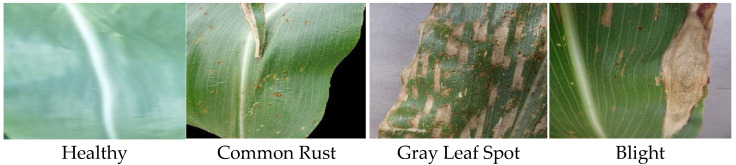
Sample images from the corn leaf disease dataset.

To achieve a deeper evaluation of the performance of the proposed method, we conducted experiments on the corn leaf disease dataset and further compared the accuracy with the previous work assessed on this dataset. The corn leaf disease dataset is part of the plant leaf disease dataset. As depicted in Figure 6, the dataset has 4188 images in total and comprises four classes including healthy corn, corn with common rust, corn with gray leaf spot, and blight. We randomly split the data into 80% for training and 20% for the evaluation stage.

### 4.2. Implementation Details

In order to evaluate our proposed approach, we compared our scheme with the existing work using the cocoa bean dataset and the corn leaf disease dataset. In addition, to give more objective results regarding the performance of the proposed approach evaluated on the cocoa bean dataset, we also compared our model with the state-of-the-art deep learning models. ResNet-101, ResNet-50, ResNet-18, and MobileNet V3 architectures were used to compare with our proposed scheme. Furthermore, for the assessment of the generalization capability, we compared the accuracy of the proposed scheme performance using public datasets.

All models were trained using the PyTorch 1.4 framework run on a personal computer (PC) with an Intel Xeon processor, 16 GB RAM, and NVIDIA 1080Ti. The deep learning models were trained from scratch without implementing any transfer learning technique. The SGD optimizer was applied to perform the optimization. In addition, the image was also resized to 224 × 224 to fit into the input size of our deep learning models. We trained the proposed model using batch size of 128, and the learning rate was initiated at 0.01 and decayed by 5 × 10^−4^.

To assess the performance of each deep learning model, we measured the inference time in the edge computing platform, Jetson Nano. PyTorch 1.4 was installed in Jetson Nano and used to run the calculation to obtain the inference result. Furthermore, we counted the computational time to produce the results from each model. We also measured the inference time of each model using the PC, as stated above.

Eventually, to give better insight related to the measurement of the inference time, we compared the computational times produced by all models using the edge computing platform (Jetson Nano) and PC. The evaluation of the deep learning performance also included the assessment of the accuracy by splitting the dataset for training and testing.

### 4.3. Evaluation Results on the Cocoa Bean Images

The deep learning schemes in this study were implemented using Python 3.6 and PyTorch 1.4 to produce the results. As seen in Table 2, the accuracy of our approach surpasses the ResNet50 and MobileNet V3. The compressed version of PCENet can achieve 88.4%, and the accuracy of the compressed version can even surpass the previous version [1,6]. As indicated in Table 2, the PCENet implemented in our previous work [1] was also beaten by the compressed model as it only reached 86.8% accuracy. Compared to the existing deep learning models, our approach outperforms ResNet101, ResNet50, and MobileNet V3.

From the model size point of view, our approach is lighter than ResNet50, while the accuracy is better than ResNet50. The model size of ResNet50 is around 188 MB, while the model size of PCENet without compression is 240 MB, and the model size of the compressed PCENet is 164 MB.

The number of parameters can also illustrate the representation of the model size. As shown in Table 2, ResNet50 has 23.52 million parameters, and our approach is only 20.43 million parameters. However, as shown in Table 2, our approach can even outperform ResNet50 even though the parameters are less than ResNet50.

To better understand our technique, we performed more pruning into PCENet. The model with a higher compression rate has only 5.12 million parameters, as indicated in Table 3. Even though more filters are pruned, we still can maintain the accuracy of 86.1%. Compared to the existing deep learning models such as ResNet18 and MobileNet V3, the accuracy of our model with the greatest compression rate is still higher than that achieved by ResNet50 and MobileNet V3.

Although the MobileNet V3 parameters are fewer than the parameters of our approach, as depicted in Table 3 (MobileNet V3 has 4.21 million and PCENet with a higher compression rate has 5.12 million), the inference time of our approach is lower than MobileNet V3: the inference time of our approach is only 0.007 s and MobileNet V3 is 0.01 s, as shown in Table 4. In addition, compared to the inference time of ResNet18, our approach has a similar inference time. However, the accuracy of our approach, which reaches 86.1%, is higher than that achieved by ResNet18, which only attains 82.7%.

### 4.4. Deployment into an Edge Platform

**Figure 7:**
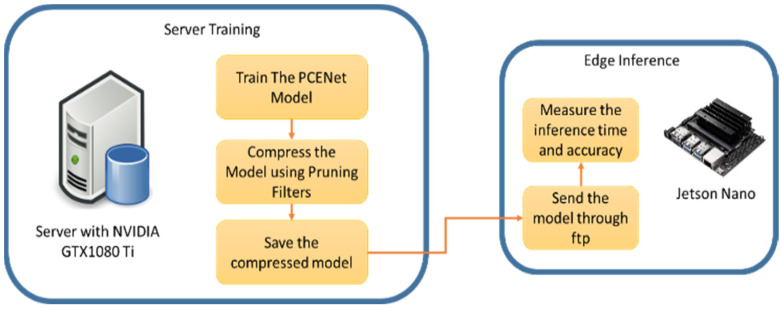
Deployment scheme from server to the edge platform.

To deploy and assess the models into an edge platform, we performed the scheme illustrated in Figure 7. The training was completed on the PC with the Ubuntu 16.04 operating system and NVIDIA 1080Ti. After training, the models were pushed and deployed into Jetson Nano using ftp (file transfer protocol). Once the ftp transfer was done, the inference stage was performed to evaluate the computational time on the edge platform. As described in Table 5, the computational time of our approach in Jetson Nano is only 0.06 s as it is lower than the existing PCENet, which is 0.101 s.

### 4.5. Assessment Results on the Corn Leaf Disease Dataset

To obtain a better understanding related to the accuracy of the methodologies on the other classification task, we conducted the evaluation using the corn leaf disease dataset [18] as a public dataset. The measurement of the performance using the public dataset is also to confirm the generalization of the proposed methodologies.

**Figure 8:**
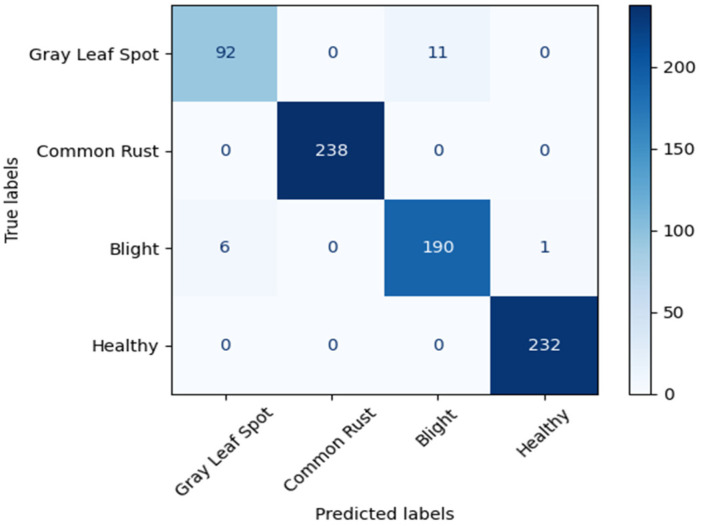
Confusion matrix of the original PCENet.

**Figure 9:**
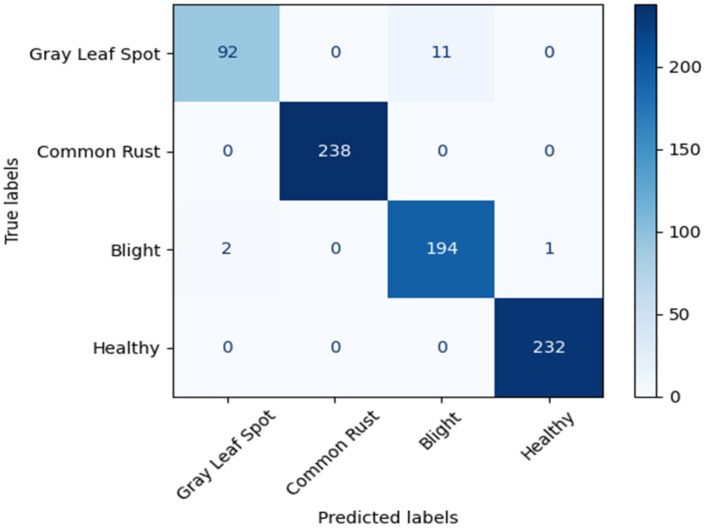
Confusion matrix of the PCENet with 25% filter reduction.

**Figure 10:**
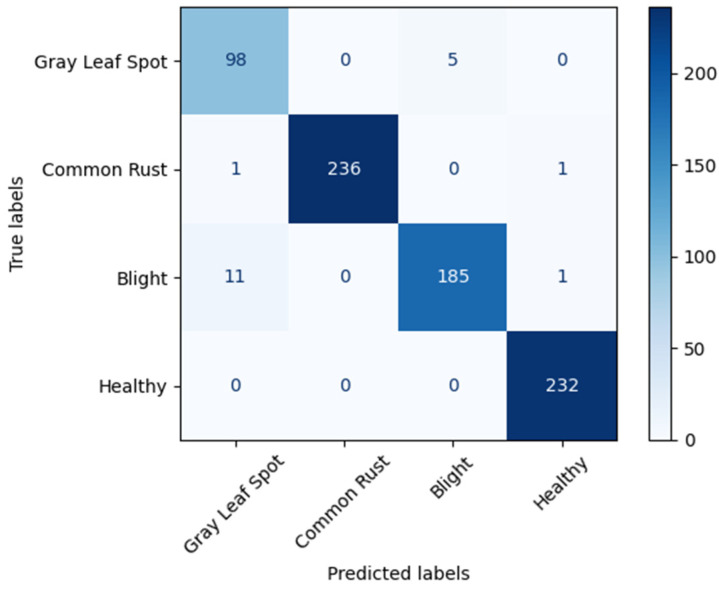
Confusion matrix of the PCENet with 50% filter reduction.

The performance of the original PCENet model is shown in Figure 8, while the proposed model is depicted in Figure 9 and Figure 10 as the confusion matrix. Furthermore, from the confusion matrix, we calculated the accuracy of each model and compared it with the accuracy of the previous work, as presented in Table 6. Surprisingly, the compressed PCENet with 25% reduction can enhance the performance of the original PCENet by achieving 98.2% accuracy. This phenomenon is consistent with the result obtained from the evaluation using the cocoa bean dataset, while the reduction of the filter by 25% increases the accuracy of the original PCENet model, as shown in Table 6.

### 4.6. Discussion

The objective of the study to accelerate the deep learning model implemented for smart farming tasks can be achieved by the proof from the data shown in the previous section. Using the pruning filter strategy, we can accelerate the existing PCENet model from 9.9 FPS to 16.7 FPS, while the most compressed model’s accuracy can still be maintained at 86.1%. Interestingly, the compression technique of pruning 25% filters gains more accurate performance than the original one; the model with 25% filters pruned achieves 88.4%, while the original PCENet accuracy is 86.8%. This phenomenon is also presented in the study of [7] and further confirmed by evaluating the compressed model on the corn leaf disease dataset by achieving an accuracy of 98.2%, while the original model only achieves 97.7% accuracy. At the same time, this might indicate that pruning filters can enhance the performance of deep neural networks by eliminating the unused filters that can cause overfitting. Using the pruning filter strategy, we can maximize the available filters while reducing the insignificant number of filters that might not contribute to producing representative features. However, a loss of the information occurred when we pruned 50% of the filters. This is indicated by the reduced accuracy experienced by the model compressed by pruning 50% of the filters. The reduction of the accuracy occurred on the experiment either assessed on the cocoa bean dataset or corn leaf disease dataset, as shown in Table 3 and Table 6.

The model size reduction undoubtedly causes the acceleration of the PCENet from 9.9 FPS to 16.7 FPS. The acceleration of the inference time is the impact of the reduction of the parameters involved to construct the deep learning model. As shown in Table 3, the original PCENet model is constructed by 29.99 million parameters. By implementing pruning filters, we can further decrease the parameters to 5.12 million, while the accuracy of 86.1% and 97.5% can be maintained for the cocoa bean classification and the corn leaf disease datasets, respectively. Therefore, the complex calculation can be reduced by lessening the number of filters with fewer contributions to generating useful features. Then, the speed of the inference time can be increased, particularly on the edge platform that is not as powerful as using the platform server or PC with dedicated GPUs.

Therefore, the FPS of each model could also be calculated and is presented in Table 5. The FPS of our compressed model is around 16.7 in Jetson Nano. ResNet18 has the same FPS in 16.7, and the FPS of MobileNet V3 is only 10.7, indicating that MobileNet V3 is slower than ours. From the data presented in Table 5, we can prove that our methodology can accelerate the existing PCENet from 9.9 FPS to 16.7 FPS while still achieving 86.1% accuracy.

## 5. Conclusions

In this research, we have proposed an alternative scheme to build a deep learning model by optimizing the existing one. Our proposed model can achieve accuracy of up to 86.1% applied to the smart farming applications specifically implemented on the classification of cocoa bean images, while the accuracy measurement of the classification of corn leaf disease images is 97.5%. In addition, our proposed model also accelerates the existing model from 9.9 FPS to 16.7 FPS, running on Jetson Nano as the edge computing platform. The computational time of our proposed scheme also outperforms the state-of-the-art models, such as MobileNet V3, while reaching the same FPS and achieving more accurate performance compared to ResNet18. Using our strategy, we can prove that a compression technique can reduce computational time and maintain acceptable and accurate performance.

Furthermore, this study can unlock the potential of the PCENet proposed in our previous work. Moreover, more research to provide more acceleration is necessary, such as applying more compression techniques and constructing a new deep learning model. Knowledge distillation or weight optimization can be used to further assess more compression schemes. Therefore, more alternatives and studies can be developed to create a smart farming application to be applied in the real world.

## Figures and Tables

**Table 1:** Dimension comparison of each layer before and after pruning.

Representation	Original Dimension	Pruning 25% Dimension	Pruning 50% Dimension
$F^{1}$	256 × 56 × 56	192 × 56 × 56	128 × 56 × 56
$F^{2}$	512 × 28 × 28	384 × 28 × 28	256 × 28 × 28
$F^{3}$	1024 × 14 × 14	768 × 14 × 14	512 × 14 × 14
$F^{4}$	2048 × 7 × 7	1536 × 7 × 7	1024 × 7 × 7

**Table 2:** Accuracy comparison among several deep learning models.

Deep Learning Architecture	Accuracy	Model Size (MB)	Parameters (Million)
ResNet101	85.3%	341	45.51
ResNet50	84.9%	188	23.52
MobileNetV3	85.7%	34	4.21
PCENet	86.8%	240	29.99
**Compressed PCENet (Reduced 25%)**	**88.4%**	**164**	**20.43**

**Table 3:** Greater compression rate on PCENet.

Deep Learning Architecture	Accuracy	Model Size (MB)	Parameters (Million)
ResNet101	85.3%	341	45.51
ResNet50	84.9%	188	23.52
ResNet18	82.7%	112	13
MobileNetV3	85.7%	34	4.21
PCENet	86.8%	240	29.99
**Compressed PCENet (Reduced 25%)**	**88.4%**	**164**	**20.43**
**Compressed PCENet (Reduced 50%)**	**86.1%**	**41**	**5.12**

**Table 4:** Inference time assessment on the server.

Deep Learning Architecture	Inference Time (Seconds)
ResNet101	0.014
ResNet50	0.009
ResNet18	0.007
MobileNetV3	0.01
PCENet	0.011
**Compressed PCENet (Reduced 25%)**	**0.008**
**Compressed PCENet (Reduced 50%)**	**0.007**

**Table 5:** Inference time assessment on the edge platform.

Deep Learning Architecture	Inference Time (Seconds)	FPS
ResNet101	0.183	5.5
ResNet50	0.086	11.6
ResNet18	0.06	16.7
MobileNetV3	0.092	10.9
PCENet	0.101	9.9
**Compressed PCENet (Reduced 25%)**	**0.077**	**13.0**
**Compressed PCENet (Reduced 50%)**	**0.06**	**16.7**

**Table 6:** Proposed methodology comparison with previous work on the corn leaf disease dataset.

Methodology	Accuracy
VGG19 + K means [19]	93.4%
LSDNet [20]	95.4%
PCENet	97.7%
**Compressed PCENet (Reduced 25%)**	**98.2%**
**Compressed PCENet (Reduced 50%)**	**97.5%**

## Data Availability

Not applicable.
